# Do Marmorkrebs, *Procambarus fallax* f. *virginalis*, threaten freshwater Japanese ecosystems?

**DOI:** 10.1186/2046-9063-8-13

**Published:** 2012-06-27

**Authors:** Zen Faulkes, Teresa Patricia Feria, Jesús Muñoz

**Affiliations:** 1 Department of Biology, The University of Texas-Pan American, Edinburg, TX, USA; 2 Real Jardín Botánico (RJB-CSIC), Plaza de Murillo 2, 28014, Madrid, Spain; 3 Universidad Tecnológica Indoamérica, Bolívar 2035 y Quito, Ambato, Ecuador

## Abstract

**Background:**

One marbled crayfish, Marmorkrebs, *Procambarus fallax* f. *virginalis* (Hagen, 1870), was discovered in a natural ecosystem in Japan in 2006. Because Marmorkrebs are parthenogenetic, they could establish a population from only a single individual, and thus pose a risk for becoming established in Japan, as they have in other countries. There are two major reasons to be concerned about the possibility of Marmorkrebs establishing viable populations in Japan. First, Japan’s only endemic crayfish, *Cambaroides japonicus* (De Haan, 1841), lives throughout Hokkaido and is endangered. Introduced Marmorkrebs are potential competitors that could further threaten *C. japonicus*. Second, Marmorkrebs live in rice paddies in Madagascar and consume rice. Marmorkrebs populations could reduce rice yields in Japan.

**Results:**

We created five models in MaxEnt of the potential distribution of Marmorkrebs in Japan. All models showed eastern Honshu, Shikoku and Kyushu contain suitable habitats for Marmorkrebs. Hokkaido, the main habitat for *C. japonicus*, contained much less suitable habitat in most models, but is where the only Marmorkrebs in Japan to date was found.

**Conclusions:**

Marmorkrebs appear to be capable of establishing populations in Japan if introduced. They appear to pose minimal threat to *C. japonicus*, but may negatively affect rice production.

## Background

In 2006, an unusual crayfish was collected in a river near Sapporo, and brought to the Sapporo Salmon Museum [1]. It had distinctive marbled colours, and all the offspring from this individual were later found to be female. This indicated that this crayfish was Marmorkrebs, a parthenogenetic [2,3] crayfish that has been provisionally identified as *Procambarus fallax* f. *virginalis* (Hagen, 1870) [4]. (Here, we refer to the parthenogenetic form as “Marmorkrebs” and the sexual form as “*P. fallax*”).

Marmorkrebs are unusual crayfish in two regards: they are the only known obligate parthenogenetic crayfish [2,3], and the only known populations in natural ecosystems are the result of human introductions. Marmorkrebs have been introduced and established populations in Madagascar [5,6] and Europe [7-10]. The discovery of Marmorkrebs in Hokkaido was the first well-documented case of an individual living in a natural ecosystem in Asia. Other reports of Marmorkrebs in Asia [11] have never been documented in the scientific literature or mainstream press. The most likely source of the Sapporo Marmorkrebs is a release or escape of a pet crayfish, as Marmorkrebs are widely circulated among aquarium hobbyists [12]. Marmorkrebs are parthenogenetic, and therefore a single female can initiate a stable population, resulting in an unwanted non-indigenous species.

In Europe, single individual Marmorkrebs were discovered years before established populations were discovered [11,13,14]. Indeed, the delay was so long that it was questioned whether Marmorkrebs could establish populations in Europe [15]. This was a reasonable hypothesis, given that not all introduced species establish populations [16]. As it happened, Marmorkrebs have established populations in Germany [7], indicating that there was a “lag phase” [17] of several years between discovery of single individuals and establishment of populations. Thus, the discovery of the Sapporo Marmorkrebs may be a precursor to finding established populations of Marmorkrebs in Japan.

We made quantitative models to assess the potential distribution of Marmorkrebs in Japan. There are at least two reasons to make such a threat assessment. First, Japan has only one native crayfish species, *Cambaroides japonicus* (De Haan, 1841). The historic range of *C. japonicus* is Hokkaido and the northern regions of Honshu [18]. This species is endangered [19], due in part to the introduction of the North American crayfish species, *Pacifastacus leniusculus*[20-24]. The introduction of another exotic crayfish species could harm the remaining *C. japonicus* populations. Second, rice farming in Japan is economically important. Marmorkrebs have damaged rice paddies in Madagascar [14], although the extent of damage is not clear [5]. Marmorkrebs could become an agricultural pest in Japan if they become established. Thus, these models may help guide monitoring efforts, policy, and public information campaigns that could prevent further introductions or limit the spread.

There are three common approaches to modeling the potential distribution of an exotic species: 1) extrapolate from the distribution of native populations only to the region of interest; 2) extrapolate from introduced populations in other regions to the region of interest; 3) extrapolate from a combination of native and introduced populations to the region of interest. Each of these methods has pros and cons, and it is not advisable to put too much weight on any single model [25].

The first, and probably the most common approach, for developing models of the potential distribution of an exotic species is to describe the native distribution of the species, and then extrapolate from the climatic variables associated with those regions to find other geographic areas that have similar climatic features [25,26]. Strictly speaking, it is not possible to use this method with Marmorkrebs because there are no known populations in the wild. We used the native distribution of *P. fallax* as a proxy for the native distribution of Marmorkrebs. We do so recognizing that, because the biotic conditions of each past introduction differ in overlap, the final fundamental niche space will vary depending on what dataset of presences is used to train the models [25]. As found in other studies of other species [27], invasive Marmorkrebs have a greater climatic range than wild *P. fallax*. There are many cases where parthenogens have quite distinct abilities and distributions than their sexual progenitors. This problem could be amplified by variation among clonal genotypes, which could be very distinct subsets of the range of abilities in the native sexual range. This has not been studied on Marmorkrebs yet, and using data from sexual *P. fallax* is a pragmatic approach.

Other approaches to modeling potential distribution are to use established populations outside the native range, and a combination of the native and introduced populations. We use all three approaches (similar to [28]). We also created two additional models that included the location of the one Marmorkrebs individual discovered in Sapporo. These models including the Sapporo Marmorkrebs are speculative, because single individuals may not indicate that there is an established or even viable population [25]. Nevertheless, if it becomes apparent that there are established populations Marmorkrebs in Japan rather than single individuals, these models may help adjust the assessment of risk accordingly.

## Results

**Figure 1:**
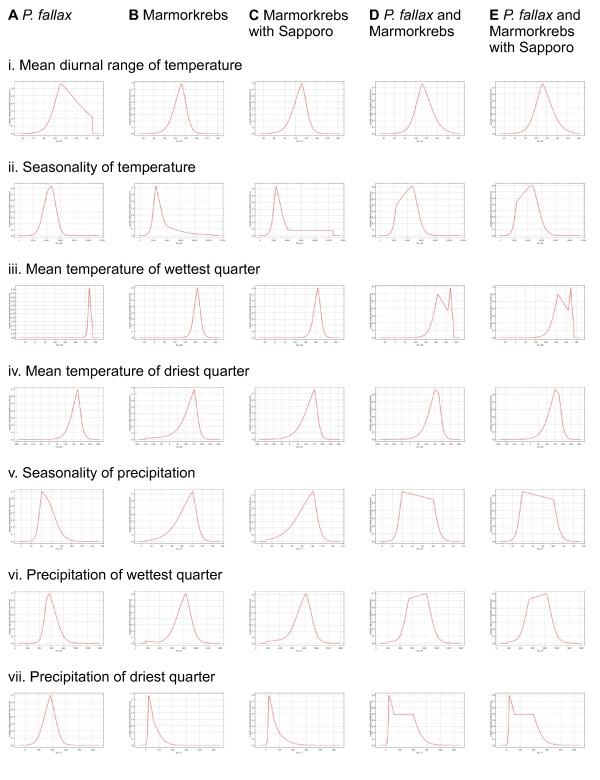
**Response curves of MaxEnt models.** Response curves of MaxEnt models created using only the corresponding variable for (**A**) *P. fallax*; (**B**) Marmorkrebs in Madagascar and Germany; (**C**) Marmorkrebs in Madagascar, Germany, and Sapporo; (**D**) *P. fallax* and Marmorkrebs in Madagascar and Germany (**E**) *P. fallax* and Marmorkrebs in Madagascar, Germany, and Sapporo. Note that the variables used in this study are not correlated, and thus the graphs reflect the dependence of predicted suitability on the selected variable. The inclusion of the individual Marmorkrebs found in Sapporo has virtually no effect on the response curves (compare column **B** to **C**, or column **D** to **E**). Wild *P. fallax* and parthenogenetic Marmorkrebs show different optimal responses to variables, except for mean temperature of driest quarter. Such different responses would explain the bimodal curves of mean temperature of wettest quarter, the hump-shaped curves of precipitation seasonality or precipitation of wettest quarter, or the asymmetrically humped curves of precipitation of driest quarter in rows **D** and **E**.

**Figure 2:**
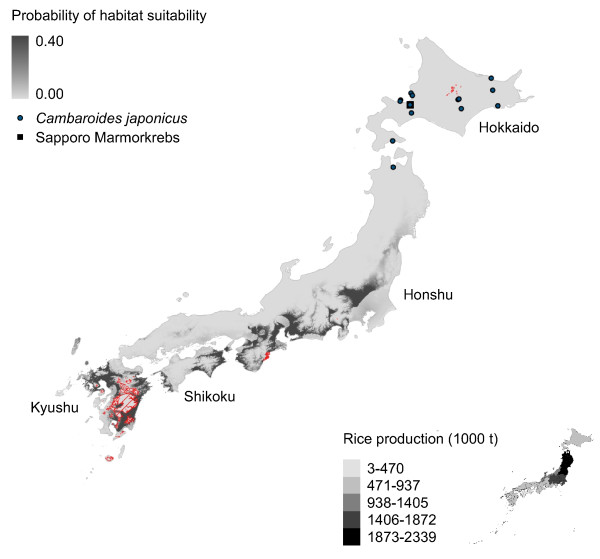
**Marmorkrebs distribution in Japan modeled using*P. fallax.*** Potential distribution of Marmorkrebs in Japan as predicted by a model trained using native *P. fallax* distribution in United States. Legend in this and subsequent figures: Gray scale indicates similarity between known and predicted habitats (i.e., habitat suitability). Circles show presence of *Cambaroides japonicus* (see Table 3); square shows location of single Marmorkrebs found in Sapporo in 2006 [1]. Regions with red hatching in this and subsequent figures show areas with novel conditions compared to the training dataset according to multivariate environmental similarity surfaces (MESS) analysis. Inset in this and subsequent figures: Choropleth map of rice production of agricultural regions [30].

**Figure 3:**
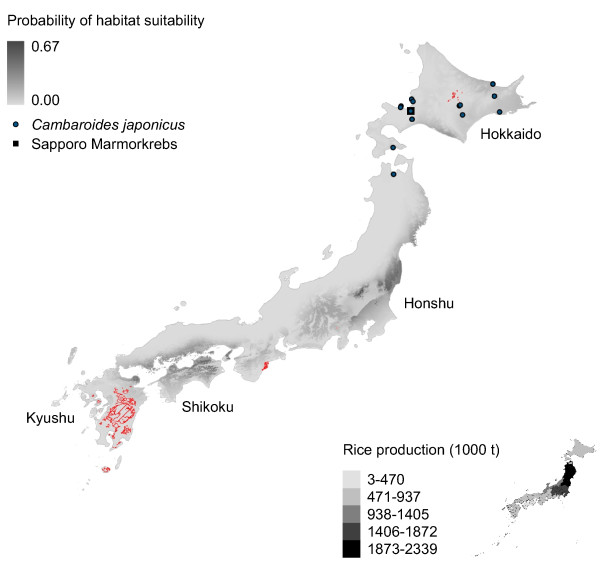
**Marmorkrebs distribution in Japan modeled using introduced Marmorkrebs.** Potential distribution of Marmorkrebs in Japan as predicted by a model trained using distributions of introduced Marmorkrebs populations in Madagascar and Europe.

**Figure 4:**
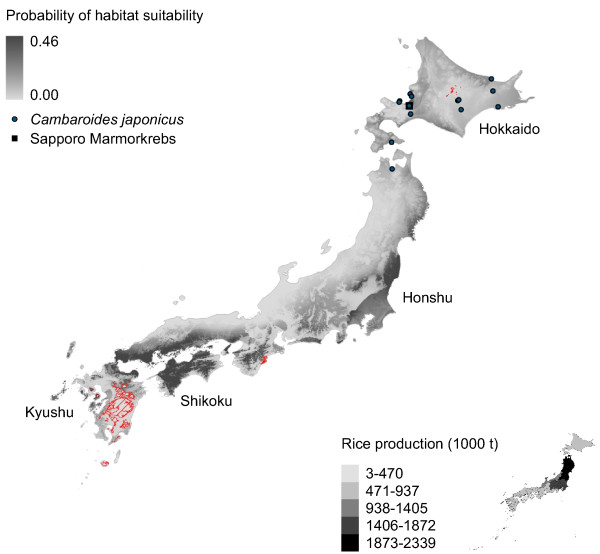
**Marmorkrebs distribution in Japan modeled using introduced Marmorkrebs and Sapporo Marmorkrebs.** Potential distribution of Marmorkrebs in Japan as predicted by a model trained using introduced Marmorkrebs populations in Madagascar and Europe, and the single individual Marmorkrebs found in Hokkaido.

**Figure 5:**
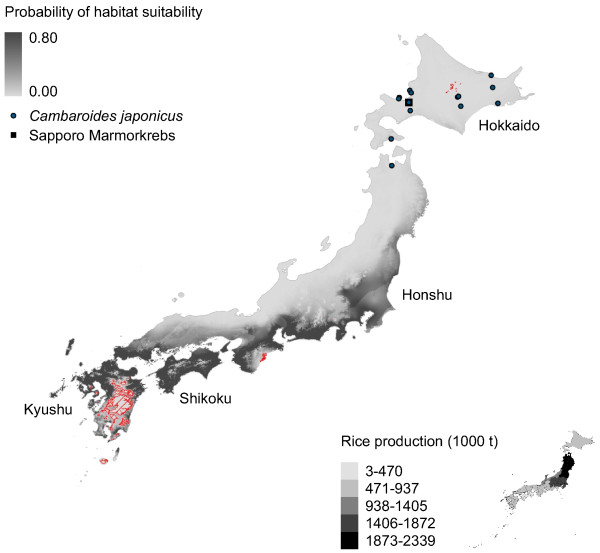
**Marmorkrebs distribution in Japan modeled using*P. fallax*and introduced Marmorkrebs.** Potential distribution of Marmorkrebs in Japan as predicted by a model trained using native *P. fallax* distribution in United States and introduced Marmorkrebs populations in Madagascar and Europe.

**Figure 6:**
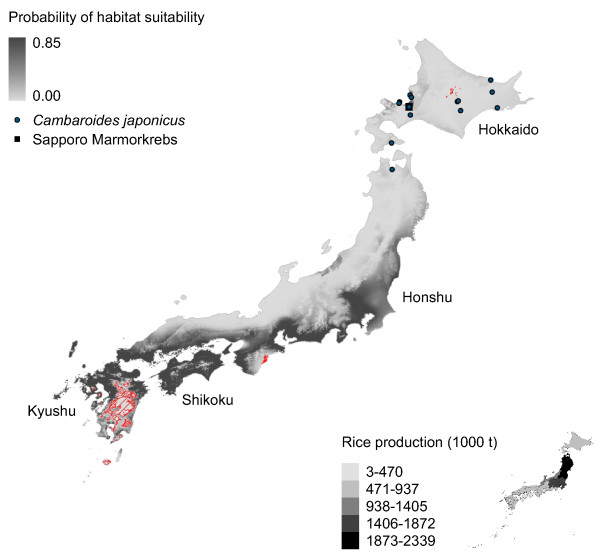
**Marmorkrebs distribution in Japan modeled using*P. fallax*, introduced Marmorkrebs, and Sapporo Marmorkrebs.** Potential distribution of Marmorkrebs in Japan as predicted by a model trained using native *P. fallax* distribution in United States, introduced Marmorkrebs populations in Madagascar and Europe, and the single individual Marmorkrebs found in Sapporo.

According to the complexity of the models, measured as the number of parameters and lower AICc values (Table 1), and the visual inspection of response variables (Figure 1), we based all further analyses on models with a regularization multiplier of 1.5 and the reduced dataset including seven uncorrelated bioclimatic variables. The multivariate environmental similarity surfaces (MESS) showed that only a fraction of central Kyushu has novel conditions compared to the training dataset (hatched areas in Figures 2, 3, 4, 5 and 6), and thus only projections in that reduced area should be considered with caution. *Procambarus fallax* wild populations and Marmorkrebs have different environmental requirements, as shown by a Mann–Whitney *U* test (Table 2) and the response curves in Figure 1.

**Table 1:** Overview of MaxEnt models

**Trained on**	**Sample size**	**beta**	**Threshold**	**Parameters**	**AICc score**	**Training AUC**	**Test AUC (30%)**
*Procambarus fallax*	37	1.0	MTSPS = 0.284	12	874.2029424	0.9773	
*Procambarus fallax*	37	1.5	MTP = 0.284	12	903.8337866	0.9764	0.9766 (0.0043)
Marmorkrebs populations	23	1.0	MTSPS = 0.033	22	not applicable	0.9844	
Marmorkrebs populations	23	1.5	MTP = 0.014	18	760.5960693	0.9813	0.9823 (0.0146)
Marmorkrebs populations and Sapporo individual	24	1.0	MTSPS = 0.045	26	not applicable	0.9869	
Marmorkrebs populations and Sapporo individual	24	1.5	MTP = 0.030	23	not applicable	0.9836	0.9723 (0.0212)
*P. fallax* and Marmorkrebs populations	58	1.0	MTSPS = 0.328	39	1829.92309	0.9673	
*P. fallax* and Marmorkrebs populations	58	1.5	MTP = 0.027	27	1660.981878	0.9623	0.9595 (0.0119)
*P. fallax*, Marmorkrebs populations, and Sapporo individual	59	1.0	MTSPS = 0.321	37	1882.800434	0.9640	
*P. fallax*, Marmorkrebs populations, and Sapporo individual	59	1.5	MTP = 0.020	24	1722.570932	0.9586	0.9537 (0.0148)

Training AUC is the AUC obtained in the 1-replicate run used for model selection and MESS generation. Test AUC is the average AUC (and standard deviation) obtained in 10 runs that used a 30% of the presence points to calculate this statistic; only models considered superior based on reduced complexity without training AUC reduction were projected and test AUC calculated. *MTSPS* – minimum training sensibility plus specificity; *MTP* – minimum training presence; *not applicable* – AICc cannot be calculated if the model have more parameters than occurrence points [29].

**Table 2:** Mean and standard deviation of bioclimatic variables

**Climatic variable**	**Mean (s.d.) of*P. fallax*, Florida populations**	**Mean (s.d.) of Marmorkrebs populations, excluding Sapporo individual**	**Mean difference**	***U*test sig. (2-tailed)**
Mean diurnal range	121.62 (11.994)	107.78 (11.969)	13.839	***
Temperature seasonality	4799.3 (729.823)	3092.17 (1600.579)	1707.123	***
Mean temperature of wettest quarter	270.05 (2.697)	206.61 (13.836)	63.445	***
Mean temperature of driest quarter	173.27 (14.327)	131.17 (53.102)	42.096	***
Precipitation seasonality	45.27 (8.549)	86.7 (28.919)	−41.425	***
Precipitation of wettest quarter	540.62 (44.529)	720.83 (233.981)	−180.204	***
Precipitation of driest quarter	181.16 (24.443)	48.43 (43.833)	132.727	***

All models predict southeastern Japan – specifically, Shikoku, Kyushu and eastern Honshu – is suitable habitat for Marmorkrebs (Figures 2, 3, 4, 5 and 6). Models trained with *P. fallax* predict that Hokkaido, the main habitat for *C. japonicus*, is unlikely to contain suitable habitat for Marmorkrebs (Figure 2), but models that included the Marmorkrebs populations (Figures 3, 4, 5 and 6) predict that Hokkaido also contains suitable habitat. All models had high average performance scores, with an averaged Area Under the Curve (AUC) in Receiver Operating Characteristics (ROC) plots over 10 runs greater than 0.95 (Table 1).

### Model 1: Trained on *P. fallax* populations

Marginal regions of eastern Shikoku and Kyushu, and south-central Honshu are likely to contain suitable habitat for Marmorkrebs under this model (Figure 2). This model predicted Japan provides less suitable habitat for Marmorkrebs than all other models. Similarly, the *P. fallax*-trained model of potential distribution of Marmorkrebs in Madagascar also predicted much less habitat than other models, and did not predict some regions where Marmorkrebs occurred [28]. The most important variables in this model were temperature of the wettest quarter, with a 46.1% contribution, and precipitation seasonality, with a 23.3% contribution.

### Model 2: Trained on Marmorkrebs populations

Under this model, most of Shikoku is suitable for Marmorkrebs, as well as a substantial part of mainland Honshu, especially to the northeastern half north of Shizuoka Prefecture (Figure 3). Kyushu and Hokkaido show less area as suitable, mainly on the coastal regions.

The climatic variables with the greatest predictive power were the same as in the previous model, although switched. The variables in the present study cannot be compared to the equivalent model in [28], because here we have removed correlated variables.

### Model 3: Trained on Marmorkrebs populations and Sapporo Marmorkrebs

This model had the greatest variation in its predictive performance. This is probably due to most Marmorkrebs presences being located in one small geographic region in Madagascar, with a small number of outliers in Germany and Sapporo. Thus, it is expected that replicate tests of model performance would be the most susceptible to which data points are included or excluded in the training and testing phase (see Methods). The variable importance is virtually identical to the previous model, which is expected given that there is only one more presence in the training dataset.

After applying the threshold rule, the regions predicted as suitable for the establishment of Marmorkrebs by this model are basically the same as predicted by the latter (Figure 1c): most of Shikoku, northern Kyushu, and a crescent area covering the east part of Honshu from southwest to northeast, although slightly displaced to the south. The addition of the data point from Hokkaido completely switches the area predicted present in this island to the western coastal regions, while the suitable area in the east disappears.

### Model 4: Trained on *P. fallax* and Marmorkrebs populations

This model is similar to models 2–3, although the areas predicted as suitable are almost continuous across Kyushu, Shikoku, and the south half of Honshu, and have virtually disappeared from Hokkaido, except for a limited region in the southern central coast (Figure 5). The inclusion of Marmorkrebs populations from Madagascar and Germany has a dramatic effect, increasing the potential area in the southern Japan islands.

The variables now more important in model construction are precipitation seasonality, as in all previous models, and mean temperature of driest quarter. The change in the variable importance can be an artefact due to the low number of Marmorkrebs localities known, and that most of the Madagascar localities are closely clustered and thus present very similar climate. Although variables are not correlated with regard to background area, they still show a strong relationship if only the Marmorkrebs localities are considered.

### Model 5: Trained on *P. fallax*, Marmorkrebs populations, and Sapporo Marmorkrebs

This model resembles Model 3 in that the addition of the Sapporo Marmorkrebs resulted in more of Hokkaido being predicted as suitable habitat (Figure 1e). However, for the rest of the study area, this model is closer to Model 4, predicting much of Kyushu, Shikoku and south Honshu as suitable for Marmorkrebs. This suggests that the combination of both the native *P. fallax* populations and the stable Marmorkrebs populations generates a more useful model by extrapolating both the fundamental climate space of the wild *P. fallax*, which is conservative, and the expanded one of Marmorkrebs, which successfully established viable populations in regions with a broader climate space.

## Discussion

Species distribution models predict that large regions of Japan are suitable habitat for Marmorkrebs. Although the most conservative model (Model 1, trained on *P. fallax* data) predicts only a few spots in Japan as suitable, the addition of established populations of Marmorkrebs highly increased the extension across Japan. The northern island of Hokkaido contains the least suitable habitat under models using existing populations; however, the addition of the temperate localities of Europe largely increased the area of potential presence in this island. These results indicate that Marmorkrebs pose a threat to freshwater ecosystems in Japan.

Marmorkrebs may pose some threat to Japan’s endangered native crayfish, *C. japonicus,* through direct competition. Model 2 shows suitable habitat overlap with Marmorkrebs' known localities in eastern Hokkaido, Model 3 overlaps around Sapporo, and in Model 5 only central Hokkaido is not predicted as suitable, but most *C. japonicus* localities coincide with high suitability areas for Marmorkrebs. Nevertheless, there is evidence that *C. japonicus* prefers deep [31] and cold [21] water, whereas Marmorkrebs appears to prefer shallow, warm waters [7]. Thus, *C. japonicus* probably faces greater competition from another cold water crayfish species, *Pacifastacus leniusculus*, than from Marmorkrebs [20-24]. Marmorkrebs may pose a threat to *C. japonicus* if Marmorkrebs carry crayfish plague. *Cambaroides japonicus* is susceptible to crayfish plague [32], but it is not clear if crayfish plague is widespread in Japan or if it has been a factor in the decline of *C. japonicus*[22]. There is no direct evidence that Marmorkrebs carry crayfish plague, but it is plausible that Marmorkrebs could act as a vector for the disease because every North American species tested has the ability to act as a chronic carrier for the causative agent of crayfish plague, the water mold *Aphanomyces astaci*[32-34].

Rice farming appears to be a more likely impact of a Marmorkrebs introduction. The eastern coastal areas of the most productive rice region, the Tohoku agricultural region in northernmost Honshu, and most of the Kanto-Tosan region, the next most productive, are consistently predicted to be highly likely to support Marmorkrebs populations (Figures 2, 3, 4, 5 and 6).

As with models of potential habitat in Madagascar, North American, and Europe of other geographic regions [28], the model trained on *P. fallax* data makes the most conservative predictions of potential distribution. This can be due to the different requirements, or wider habitat breadth observed in Marmorkrebs (Table 2). Models that include Marmorkrebs data predict larger areas as potential habitat. There are several points to keep in mind in interpreting these models. The *P. fallax*-trained model was the most conservative in predicting the distribution of introduced Marmorkrebs, and vice versa [28], similar to what was found for the native and introduced distributions of signal crayfish in Japan [35]. Both studies interpreted these imperfect predictions indicating a niche shift for these crayfish species. The models predict lower habitat suitability in northern Japan, suggesting that the models pass the “eco-plausibility test” [25].

There are two potential limitations to the models presented here. First, the locations of *P. fallax* have uncertainty associated with them, because the locations were estimated from records at the level of counties. The abundance and apparently uniform presence of *P. fallax* throughout its range, occupying “practically all of the streams” in its range [36], however, mitigates against the uncertainty inherent in using county level records. Second, terrestrial climatic variables were used to train the models rather than variables describing the aquatic habitats. Temperature and precipitation are the two major categories of climatic variables often used in species distribution models. Terrestrial air temperature correlates with lake water temperature [37], and water temperature affects growth and mortality in Marmorkrebs [38]. Similarly, precipitation levels (i.e., drought) affect wild populations of *P. fallax*[39,40]. Thus, there are good reasons to expect that terrestrial climate variables can predict a substantial amount about the distribution of aquatic species; indeed, several other studies have done so [28,35,41-43]. Further research is needed to determine if mismatches in predicted and actual distributions of aquatic species (e.g., signal crayfish [35]) might be explained by variables about aquatic habitats being omitted from models. Currently, however, there is no database for features of freshwater bodies equivalent to that for terrestrial climate.

## Conclusion

Controlling crayfish populations in other regions, such as Europe, has proved to be extraordinarily difficult. Physical barriers, such as dams [44], may hinder the spread of introduced crayfish, but there are no control agents that can target and remove crayfish once established. Drastic non-targeted measures, such as applying biocides to entire water bodies [45], have had mixed success. As the pet trade is the most likely source of Marmorkrebs introductions, education of pet owners and promoting responsible ownership may be a cost effective measure to reduce the chance of Marmorkrebs becoming established in Japan.

## Methods

### Species distribution data

We used the distributions of established populations of *Procambarus fallax* in the United States and established populations of Marmorkrebs in Madagascar that were used previously [28] (spreadsheet of locations used in that model available at http://marmorkrebs.org). The main difference in this dataset and that used in [28] was that we removed locations in Europe where only single Marmorkrebs had been reported [11,15,46,47] and added two established Marmorkrebs populations that were subsequently found in Germany [7-9] and one new population found in Madagascar [Lake Alaotra; J.P.G. Jones, personal communication]. In all cases, the locations used to train the models represent populations of crayfish, not single individuals. Single individuals that are occasionally found outside of the normal range, but do not survive there, can distort models [25].

The distribution of *P. fallax* was taken from previously published records of its presence in American counties [36,48]. County names were searched though Google Earth v. 5.2.1. (http://www.google.com/earth/), which provided latitude and longitude measurements near the center of each county. Although the available location data may be coarse, Hobbs noted that *P. fallax* is abundant throughout most of its distribution and could be found in “practically all of the streams” in its range [36].

The distribution of Marmorkrebs in Madagascar was estimated from maps and published GPS coordinates [5,6]. Published maps were superimposed on locations in Google Earth with the “Add Image Overlay” tool. The transparency of the map was adjusted so that both the published map and the Google Earth image simultaneously. Placemarks on Google Earth were put onto points indicated on the published map, from which latitude and longitude were recorded.

The distribution of Marmorkrebs in Europe were taken from published GPS coordinates [7] or estimated from city locations [8,9] using Google Earth.

To visualize the potential overlap of Marmorkrebs and *C. japonicus* distributions, we searched the scientific literature for records of specific locations of *C. japonicus* (Table 3). In cases where GPS coordinates were not given, locations were estimated by nearest reference to a city or other geographic feature using Google Earth.

**Table 3:** **Locations of*Cambaroides japonicus*described in the scientific literature**

**Latitude**	**Longitude**	**Location**	**Reference**	**Estimation**
41.76879	140.7288	Hakodate, Hokkaido	[49]	City
43.02701	144.5133	Kushiro District, Hokkaido	[50]	City
43.25	143.0833	Lake Komadome, Hokkaido	[51]	GPS
43.23482	141.014	Otaru, Hokkaido	[52]	GPS
43.48327	141.3866	Hamamasu, Hokkaido	[52]	GPS
43.39928	141.4354	Atsuta, Hokkaido	[53]	Estimated
42.7712	141.4022	Shikotsu Lake, Hokkaido	[54]	City
43.27465	143.1141	Lake Shikaribetsu, Hokkaido ^a^	[22]	City
42.9239	143.1961	Kikanko River, Obihiro City, Hokkaido ^a^	[21]	City
40.82207	140.7474	Aomori, Honshu	[55]	City
43.59268	144.3276	Kusshara Lake, Hokkaido	[55]	Estimated
43.19072	140.9947	Otaru City, Hokkaido	[56]	City
44.02063	144.2734	Oshoppu stream in Abashiri City, Hokkaido	[57]	City
43.0621	141.3544	Sapporo, Hokkaido	[58]	City

^a^Extirpated.

### Rice production

Data on paddy field rice production for “agricultural regions” was taken from [30]. Each agricultural region is composed of one or more prefectures. We used GIS coordinates for prefectures from [59], with each prefecture being assigned the average rice production for its agricultural region.

### Modeling process

The modeling process was similar to that used in [28]. Models are trained using the existing distribution of the species of interest. The distribution of the species is matched to bioclimatic variables, and potential distributions are then predicted which finds other regions with bioclimatic conditions. Terrestrial climatic variables and those of non-ocean aquatic habitats are correlated in many ways. For example, terrestrial air temperature correlates well with water temperature in lakes [37]. Several studies have used terrestrial climatic variables to model the broad-scale distribution of freshwater aquatic species across terrestrial landscapes [28,35,41-43].

We created models using MaxEnt v. 3.3.3e [26,60] because it discriminates suitable and unsuitable areas better than other methods [26,61-63]. While some parameters were set to defaults (convergence threshold = 10–5, or maximum iterations = 500), we used two regularization multiplier values (1.0 and 1.5). A regularization multiplier of 1.5 allows that variables’ average values in the projections spread from the empirical average of the background points (the situation if it is set to 1.0), thus avoiding model overfitting [29]. The 10,000 background points were randomly selected from the areas of the immediate adjacency of presences and pertaining to the same bioclimatic region according to the Köppen-Geiger classification scheme [64-66]. To test different training strategies, we created five presence datasets: 1) the native distribution of *P. fallax* in the United States; 2) the established populations of Marmorkrebs in Madagascar and Germany; 3) the established populations of Marmorkrebs in Madagascar and Germany, plus the Sapporo Marmorkrebs; 4) a combination of the American distribution of *P. fallax* and Marmorkrebs established populations in Madagascar and Germany; and 5) the American distribution of *P. fallax*, the Marmorkrebs populations in Madagascar and Germany, plus the Sapporo Marmorkrebs.

The nineteen Worldclim bioclimatic variables (http://www.worldclim.org) were intersected with the presences and background datasets. To avoid multicollinearity and model overfitting, we ran a correlation analysis on the background dataset and eliminated one of the variables in each pair with a Pearson correlation value >0.8. The final datasets (Table 2) included mean diurnal range (mean of monthly (max temp - min temp)); temperature seasonality (standard deviation × 100); mean temperature of wettest quarter; mean temperature of driest quarter; precipitation seasonality (coefficient of variation); precipitation of wettest quarter, and; precipitation of driest quarter. These bioclimatic variables result from global land area interpolation of climate point data (years 1950–2000) at a spatial resolution of 30 arc-sec [67] which corresponds to a spatial resolution of approximately 0.008333° at the Equator (1 × 1 km^2^ grid cells).

To confirm that combinations of novel climates were not cause of concern in the projections, we follow [68,69] to generate the multivariate environmental similarity surfaces (MESS); this grid was reclassified and values below zero were masked to show areas of novel climate space relative to the range under which the model was fitted.

To decide the combination of parameters that would be projected onto the final model, we used the small sample corrected Akaike’s Information Criterion (AICc) implemented in ENMTools [29]. The less complex model, i.e. lower AICc, was projected to the globe at 5 km pixel size, and to Japan at 1 km pixel size. To generate the presence/absence grids, we used the “minimum training presence” threshold, as in similar studies on invasive species [25]. For further testing of model transferability, we also checked the shape of the response curves looking for open-ended, unrealistic responses (Figure 1).

To evaluate the predictive ability of these models, ten replicates of each model were run. In each replicate, 70% of the locations with known occurrences were selected randomly to train the model. The final models were in all cases the average of their 10 replicates for each set of training data; and the threshold used to create the binary grid the average threshold over the runs. The accuracy of each model was estimated using the Area Under the Curve (AUC) in Receiver Operating Characteristics (ROC) plots [70], calculated in MaxEnt on the 30% unused presences. ROC is a threshold–independent measure that evaluates the probability that the model produces a positive result in a positive locality (sensitivity) versus the probability that the model produces a negative result in a negative locality (specificity) when presented with new data. A ROC plot is obtained by plotting all sensitivity values on the y–axis against their equivalent (1–specificity) values for all available decision thresholds on the x–axis. An AUC score of 1 indicates perfect performance; a score of 0.5 indicates random performance.

## Competing interests

The authors declare no competing interests.

## Authors’ contributions

ZF compiled data; TPF and JM ran models; ZF, TPF, and JM wrote the paper. All authors read and approved the final manuscript.
